# Supplementary material for: Long-Term Consequences of Asymptomatic SARS-CoV-2 Infection: A Systematic Review and Meta-Analysis
Source: Int J Environ Res Public Health. 2023 Jan 16;20(2):1613. doi: 10.3390/ijerph20021613 (PMC9863678; doi:10.3390/ijerph20021613)
Supplement: Supplementary file 1 [file ijerph-20-01613-s001.zip › supplementary-Table S2.pdf]

Supplementary table S2. Subgroup analysis for long-term consequences of asymptomatic and symptomatic SARS-CoV-2 infection based on the object of study

Consequences	Asymptomatic infection n/N	Symptomatic infection n/N	OR	95%CI	P-value	I^2 (%)	P-Heterogeneity
**At least one symptom**							
Children	56/444	116/255	0.25	0.08-0.81	<0.05	76.90	<0.05
Adults	20/99	460/786	0.13	0.02-1.21	>0.05	90.60	<0.05
Overall	76/543	576/1041	0.20	0.09-0.45	<0.05	79.40	<0.05
**Fatigue**							
Children	11/444	43/255	0.18	0.03-1.15	>0.05	80.90	<0.05
Adults	8/99	169/786	0.27	0.12-0.59	<0.05	0.00	>0.05
Overall	19/543	212/1041	0.19	0.08-0.49	<0.05	61.30	>0.05
**Cutaneous lesions**							
Children	3/399	9/230	0.19	0.05-0.69	<0.05	-	-
Adults	1/99	27/786	0.43	0.08-2.27	>0.05	0.00	>0.05
Overall	4/498	36/1016	0.26	0.09-0.72	<0.05	0.00	>0.05
**Dyspnoea**							
Children	2/45	4/25	0.24	0.04-1.44	>0.05	-	-
Adults	3/99	85/786	0.26	0.08-0.78	<0.05	0.00	>0.05
Overall	5/144	89/811	0.25	0.10-0.65	<0.05	0.00	>0.05
**Loss of taste/smell**							
Children	4/444	24/255	0.11	0.01-1.44	>0.05	75.70	<0.05
Adults	0/55	62/541	0.07	0.00-1.13	>0.05	-	-
Overall	4/499	86/796	0.10	0.02-0.58	<0.05	53.50	>0.05
